# Paradoxical Infarction? Inferior STEMI With Unexpected Discovery of ASD in an Oncology Patient: A Case of Combined Percutaneous Intervention

**DOI:** 10.1002/ccr3.9569

**Published:** 2024-12-16

**Authors:** Miguel Angel Balbuena Madera, Juan Francisco Garcia Garcia, Antonio Vargas Cruz, Heberto Aquino Bruno, Jesús Guadalupe González Jasso

**Affiliations:** ^1^ Department of Cardiology National Medical Center November 20 Mexico City Mexico; ^2^ Universidad Nacional Autonoma de Mexico Mexico

**Keywords:** acute myocardial infarction, atrial septal defect, oncology patient, paradoxical coronary artery embolism, percutaneous coronary intervention

## Abstract

This case report explores the management of a 56‐year‐old female oncology patient presenting with acute ST‐elevation myocardial infarction (STEMI) and an incidental atrial septal defect (ASD). The patient, with a history of rectal cancer and hypothyroidism, experienced acute chest pain and dyspnea. She was diagnosed with an inferior STEMI and underwent percutaneous coronary intervention (PCI) with the placement of three medicated stents in the right coronary artery. During hospitalization, an echocardiogram revealed a significant ostium secundum ASD. Angiography indicated thrombi, suggesting a potential paradoxical embolism. Percutaneous ASD closure was performed during the same hospital stay, leading to a favorable clinical course without immediate complications. This case highlights the importance of a multidisciplinary approach and comprehensive evaluation in managing complex cardiovascular conditions, particularly in patients with increased thrombotic risk due to malignancy.

## Graphical abstract

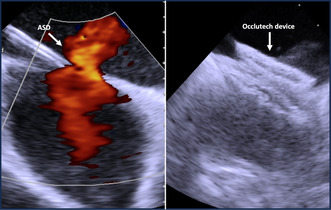

**Summary**

- Managing acute ST‐elevation myocardial infarction with a paradoxical embolism through an atrial septal defect in oncology patients is challenging.
- This case shows that combining percutaneous coronary intervention and atrial septal defect closure in one hospitalization optimizes outcomes by stabilizing hemodynamic status and preventing future thromboembolic events.

## 1 Introduction

Acute ST‐elevation myocardial infarction (STEMI) is a medical emergency requiring prompt intervention to restore coronary blood flow. The coexistence of an atrial septal defect (ASD) in patients with STEMI adds clinical complexity, particularly in those patients with a history of cancer. The prevalence of ASD in the general population is between 0.1% and 0.2%, and this anomaly can predispose to paradoxical embolisms due to the abnormal passage of thrombi through the interatrial defect [1].

Managing STEMI in the presence of previously undiagnosed ASD presents significant challenges. Comprehensive echocardiographic evaluation is crucial for detecting structural abnormalities that may influence treatment strategy and prognosis [2]. Additionally, oncology patients have an increased risk of thrombosis and paradoxical embolisms due to hypercoagulable states [3].

## 2 Case History and Examination

A 56‐year‐old female patient with a history of primary hypothyroidism and rectal cancer presented to the medical center with acute chest pain and sudden onset of dyspnea. Upon evaluation, she was diagnosed with STEMI involving the inferior region, with electrical and mechanical extension to the right ventricle. Her condition was further complicated by the development of complete atrioventricular (AV) block, requiring placement of a temporary pacemaker.

**FIGURE 1:**
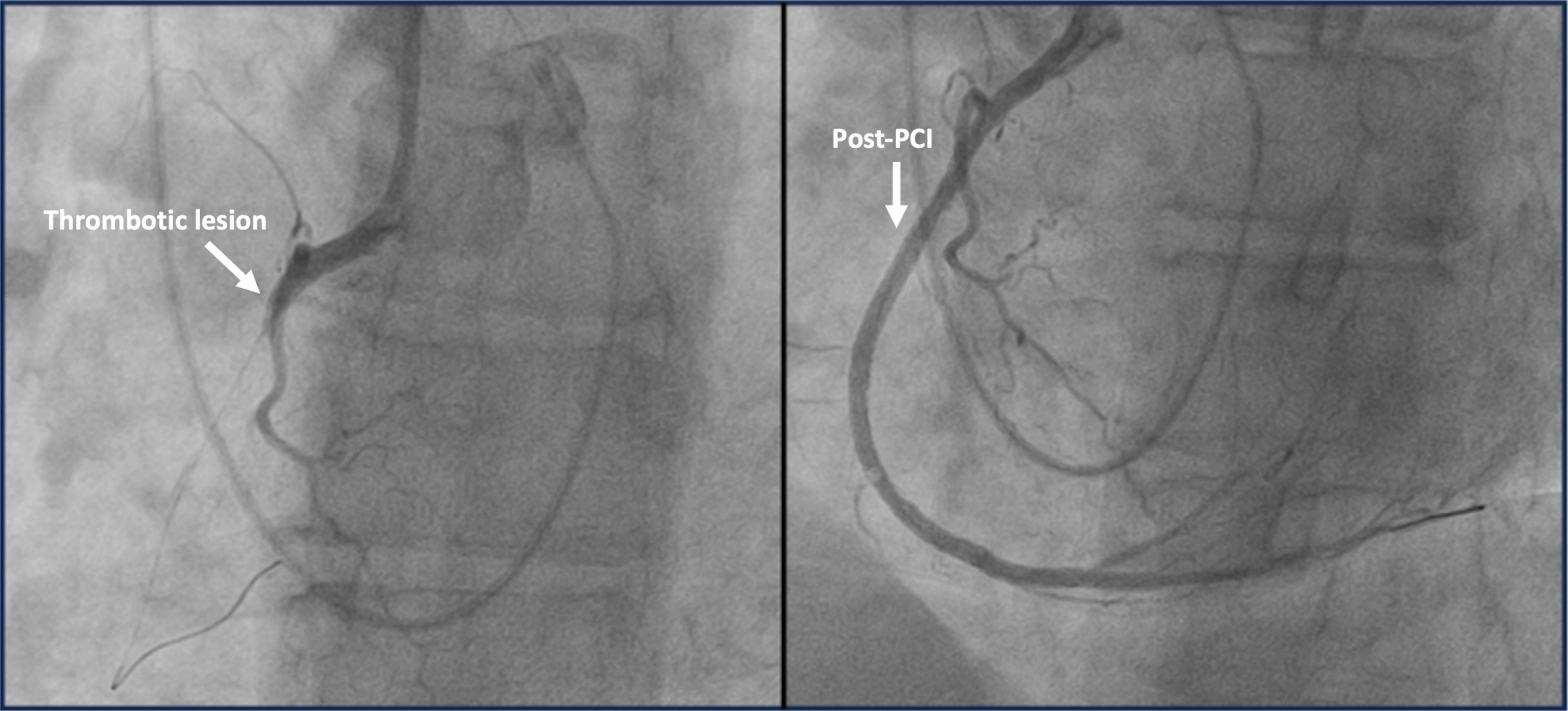
Right coronary artery with acute thrombotic lesion and angioplasty with three medicated stents.

Coronary angiography revealed an acute thrombotic occlusion in the proximal segment of the right coronary artery. Primary percutaneous coronary intervention (PCI) was performed, with the placement of three medicated stents (2.27 × 38 mm, 3.0 × 33 mm, and 2.75 × 23 mm), successfully restoring distal flow (Figure 1). Due to the high thrombus burden, a IIb/IIIa inhibitor was also administered. Despite the severity of her condition, the AV block subsided in the following days, allowing for pacemaker removal.

## 3 Methods

During hospitalization, an echocardiographic evaluation revealed the unexpected presence of an ostium secundum‐type ASD with a maximum length of 29 mm × 20 mm, an area of 4.65 cm^2^, and aortic borders of 5.6 mm, AV border of 15.5 mm, posterior border of 8 mm, superior vena cava border 12.3 mm, and inferior vena cava border 17 mm, with a significant left‐to‐right shunt. This discovery was of particular concern due to its potential hemodynamic impact and possible association with acute myocardial infarction.

The decision to proceed with ASD closure during the same hospitalization was based on the need to optimize the patient's hemodynamic status. Extension of the infarction to the right ventricle requires careful optimization of fluid preload. In the presence of an ASD, the pressure flow between the left and right atria is altered, potentially complicating hemodynamic management. In this context, ASD closure was deemed crucial to stabilize intracardiac pressures and enhance the effectiveness of AMI treatment.

**FIGURE 2:**
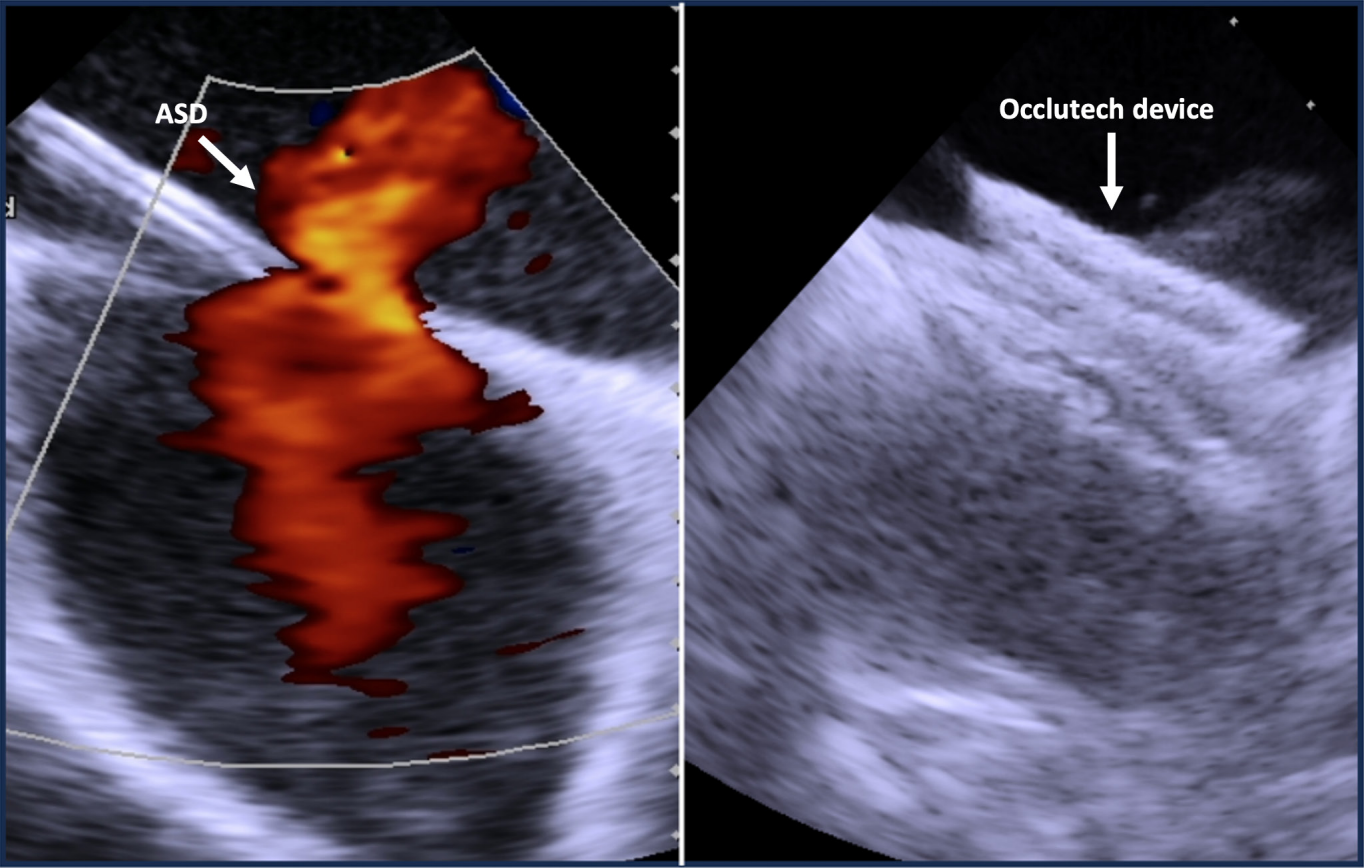
Intracardiac ultrasound evaluation of atrial septal defect and percutaneous closure with Occlutech 27‐mm.

For percutaneous ASD closure, a 27‐mm Occlutech device was used. The selection of this size was based on intracardiac echocardiographic measurements estimating the defect size at 22 mm (Figure 2). Considering margin optimization around the defect edges, an additional 5 mm was added, leading to the selection of a 27‐mm device to ensure effective and stable closure.

## 4 Outcome and Follow‐Up

**FIGURE 3:**
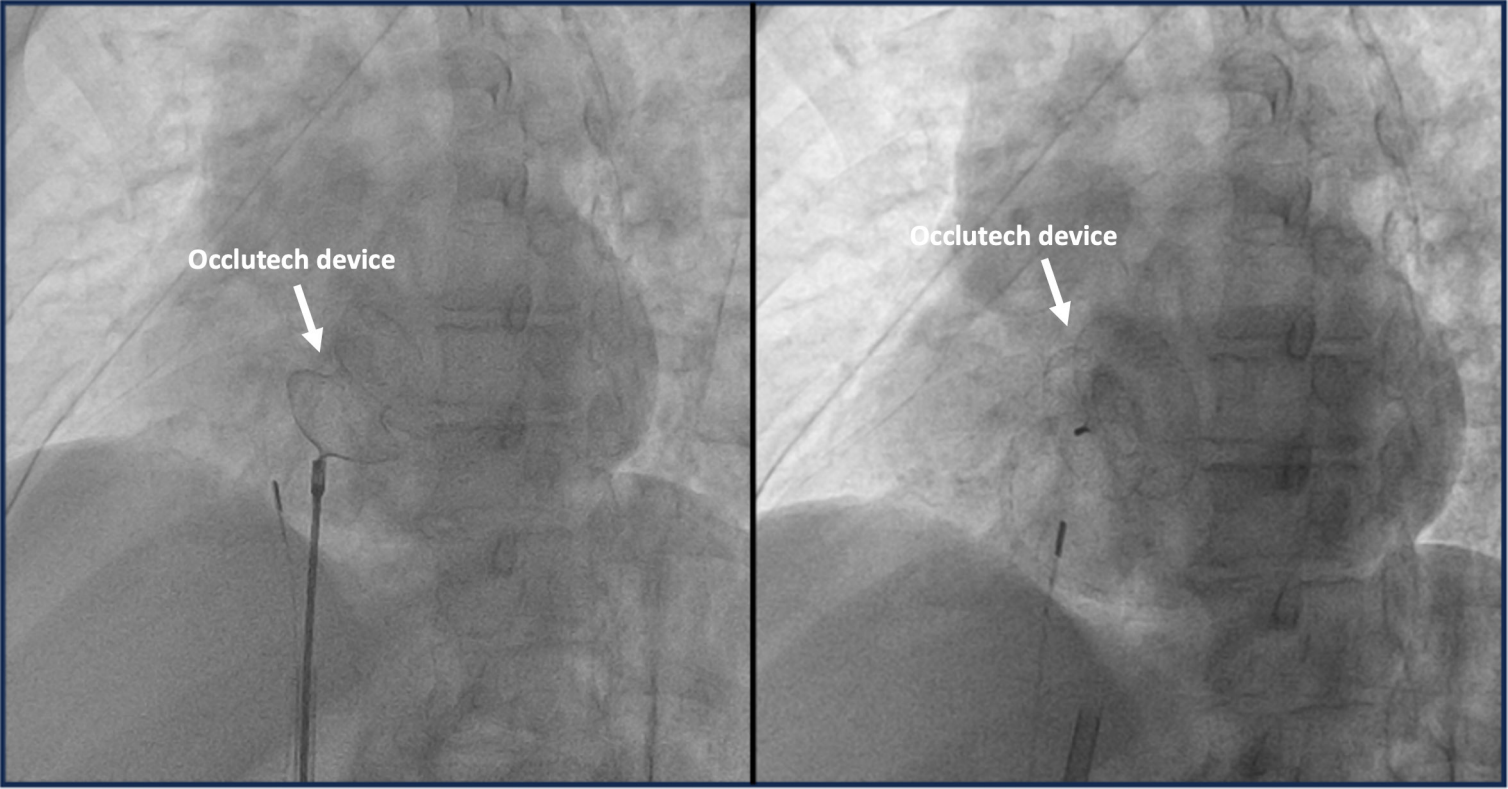
Fluoroscopy (anteroposterior view) showing proper placement of the Occlutech 27‐mm device.

The percutaneous ASD closure procedure was successfully performed, optimizing the patient's hemodynamic status and preventing potentially serious future complications. The technique involved device insertion through a catheter, guided by both echocardiographic and fluoroscopic imaging to ensure accurate and effective placement (Figure 3). The patient's clinical course was favorable following the procedure.

The comprehensive management of a complex case involving acute STEMI and incidental identification of an ASD in an oncology patient underscores the importance of a multidisciplinary and coordinated approach. PCI successfully revascularized the right coronary artery, while percutaneous ASD closure using Occlutech device, guided by precise echocardiographic measurements, optimized the patient's hemodynamic status, and prevented future paradoxical thromboembolic complications.

This case highlights the efficacy of combining PCI and ASD closure in a single hospitalization. The patient's favorable clinical course underscores how the integration of advanced techniques and interdisciplinary collaboration can significantly improve outcomes in patients with complex cardiovascular conditions. This approach should be considered in similar scenarios to optimize patient care and reduce the risk of long‐term complications.

## 5 Discussion

The combination of acute STEMI and incidental identification of an ASD presents a significant diagnostic and therapeutic challenge, especially in patients with a history of cancer. Although the prevalence of paradoxical embolism as a cause of STEMI is rare, its identification is crucial due to its implications for patient management [4, 5]. In this case, we cannot definitively confirm that the STEMI was caused by a paradoxical embolism, but this possibility cannot be ruled out, given the finding of thrombi in the angiography and the presence of the ASD.

In the context of an STEMI, the detection of an ASD requires careful risk–benefit assessment of defect closure during the same hospitalization. This strategy is supported by recent guidelines recommending percutaneous closure in patients with significant ASD and left–right shunt to prevent hemodynamic and thromboembolic complications [6, 7].

It is important to note that due to the urgent need for management during primary angioplasty, intravascular imaging, which would have been the ideal method to evaluate the presence of an atherosclerotic plaque and the characteristics of the coronary lesion, was not performed. Given the clinical scenario of an acute STEMI and an acute thrombotic occlusion, it was not feasible at that moment. The use of medicated stents was considered the best option to ensure effective revascularization and restore optimal coronary flow. The integration of coronary intervention and percutaneous ASD closure in a single hospitalization demonstrates the effectiveness of a comprehensive and coordinated approach, optimizing the patient's hemodynamic status and preventing future serious complications. This case highlights the importance of thorough evaluation and interdisciplinary collaboration in managing patients with complex cardiovascular conditions, leading to significant improvement in clinical outcomes.

## Author Contributions

**Miguel Angel Balbuena Madera:** conceptualization, investigation, project administration, writing – original draft, writing – review and editing. **Juan Francisco Garcia Garcia:** project administration, writing – review and editing. **Antonio Vargas Cruz:** conceptualization, writing – original draft. **Heberto Aquino Bruno:** conceptualization, writing – original draft. **Jesús Guadalupe González Jasso:** conceptualization, writing – original draft.

## Ethics Statement

Consent from the patient is deemed to be enough.

## Consent

Written informed consent was obtained from the patient to publish this report in accordance with the journal's patient consent policy.

## Conflicts of Interest

The authors declare no conflicts of interest.

## Data Availability

Data related to the case report can be made available on request.
